# The 300 Marines: characterizing the US Marines with perfect scores on their physical and combat fitness tests

**DOI:** 10.3389/fphys.2024.1406749

**Published:** 2024-06-18

**Authors:** David P. Looney, Adam W. Potter, Erica A. Schafer, Christopher L. Chapman, Karl E. Friedl

**Affiliations:** ^1^ United States Army Research Institute of Environmental Medicine (USARIEM), Natick, MA, United States; ^2^ Oak Ridge Institute for Science and Education (ORISE), Oak Ridge, TN, United States

**Keywords:** performance, military, standards, exercise, fitness, body composition

## Abstract

Few US Marines earn perfect 300 scores on both their Physical Fitness Test (PFT) and Combat Fitness Test (CFT). The number 300 invokes the legendary 300 Spartans that fought at the Battle of Thermopylae, which inspired high physical fitness capabilities for elite ground forces ever since.

**Purpose:**

Determine distinguishing characteristics of the “300 Marines” (perfect PFT and CFT scores) that may provide insights into the physical and physiological requirements associated with this capability. These tests have been refined over time to reflect physical capabilities associated with Marine Corps basic rifleman performance.

**Materials and methods:**

Data were analyzed from US Marines, including 497 women (age, 29 ± 7 years; height 1.63 ± 0.07 m; body mass, 67.4 ± 8.4 kg) and 1,224 men (30 ± 8 years; 1.77 ± 0.07 m; 86.1 ± 11.1 kg). Marines were grouped by whether they earned perfect 300 scores on both the PFT and CFT (300 Marines) or not. We analyzed group differences in individual fitness test events and body composition (dual-energy x-ray absorptiometry).

**Results:**

Only 2.5% (*n* = 43) of this sample earned perfect PFT and CFT scores (*n* = 21 women; *n* = 22 men). Compared to sex-matched peers, 300 Marines performed more pull-ups, with faster three-mile run, maneuver-under-fire, and movement-to-contact times (each *p* < 0.001); 300 Marines of both sexes had lower fat mass, body mass index, and percent body fat (each *p* < 0.001). The lower percent body fat was explained by greater lean mass (*p* = 0.041) but similar body mass (*p* = 0.085) in women, whereas men had similar lean mass (*p* = 0.618), but lower total body mass (*p* = 0.025).

**Conclusion:**

Marines earning perfect PFT and CFT scores are most distinguished from their peers by their maneuverability, suggesting speed and agility capabilities. While both sexes had considerably lower percent body fat than their peers, 300 Marine women were relatively more muscular while men were lighter.

“***Come and take them***.”

King Leonidas of Sparta, in response to King Xerxes of Persia’s demands to lay down their weapons at the Battle of Thermopylae.

## Introduction

In 1980, President Jimmy Carter directed the US military to review and improve fitness standards in response to a recognition that the post-Vietnam era military demonstrated an increasing rate of obesity and a decline in physical fitness (Friedl, 2004; Friedl, 2009; Friedl, 2012; Friedl, 2017; Friedl and Grate, 2019; Friedl et al., 2020; Potter et al., 2022a; Harty et al., 2022). Of all the military services, the US Marine Corps (USMC) place the highest priority on maintaining an exceptional standard for individual physical fitness and physical endurance on the battlefield (Friedl, 2017; Potter et al., 2022a). In the 1980 review, the US Marines were held up as the example for the other services for physical fitness testing and for circumference-based body fat testing and enforcement (Friedl, 2017). US Marines are amongst the most physically fit conventional military populations, and these standards have been upheld over time, while the other services have continued to modify and ease standards to meet recruiting and retention goals (Potter et al., 2022a; Potter et al., 2022b; Looney et al., 2023). This high standard of fitness is currently enforced by two annual assessments: the Physical Fitness Test (PFT) and Combat Fitness Test (CFT) (Keefer and Debeliso, 2020; Office of the Commandant of the Marine Corps, 2022). While all Marines are encouraged to earn a first class score of 235 or more on each test (Office of the Commandant of the Marine Corps, 2022), very few US Marine women and men achieve a perfect 300 score on both their PFT and CFT (Bartlett et al., 2015; Keefer and Debeliso, 2020; Keefer et al., 2021; Potter et al., 2023).

For many servicemembers, the number 300 invokes perhaps the most famous military operational unit in history: the 300 Spartans that defended against overwhelming Persian invaders at the Battle of Thermopylae (Berkey et al., 2022). The 300 Spartans have been glorified in popular culture for their exceptional heroism (Murray, 2007) and used as inspiration for peak physical and combat fitness attainment by tactical and recreational populations alike (Titus et al., 2020). With this shared numerical connotation, the “300 Marines” that achieve perfect scores on both their PFT and CFT represent a modern-day population with elite levels of fitness that other servicemembers can aspire to.

The importance of physical fitness to military readiness is well established, with benefits to performance, near term health, and to injury prevention. Overweight and underweight individuals are at higher risk for musculoskeletal injury during recruit training (Jones et al., 2017). In soldier data, poor muscle development is markedly associated with reduced strength capability and excess adiposity is associated with decrease aerobic performance (Friedl, 2004). In addition to musculoskeletal injury risk, obesity increases risk of chronic health conditions such as type 2 diabetes and cardiovascular diseases, which can impede soldiers’ ability to deploy and sustain operations (National Heart et al., 1998). A fit military appearance also serves as a powerful deterrent by projecting strength, discipline, and capability. It conveys a message of readiness and resilience to potential adversaries, deterring aggression and conflict, and it instills confidence and reassurance among civilians, reinforcing trust in the armed forces’ ability to protect and defend.

The purpose of our study was to identify defining physical characteristics of the fittest US Marine women and men. We aimed to determine which aspects of physical and combat fitness most distinguished the 300 Marines from their peers. In addition, we sought to assess just how different the body composition of these 300 Marines was from other Marines. Sexual dimorphism in human physical strength and anthropometry is well established (Bartlett et al., 2015) with certain sex-differences in performance explained by relative strength (Nimphius et al., 2019; Comfort et al., 2024). We hypothesized that the defining characteristics of a 300 Marine converged for women and men by being highly associated. Ultimately, our study would provide tactical populations with benchmark values for motivation when participating in nutritional, training, and general health interventions.

## Materials and methods

We performed a cross-sectional study on data collected over a multi-year, multi-site body composition survey of the USMC (Potter et al., 2022c) to identify defining characteristics of the most fit Marine women and men. Each sex was divided into groups: Marines that earned perfect 300 scores on both their last PFT and CFT (300); and those that did not (Other). Table 1 displays requirements to earn maximum 100 points for each USMC fitness test event by sex and age group. We tested for significant group mean differences in fitness test event performance and anthropometrics between the 300 and Other Marines. In addition, we examined how correlated these group mean differences were between Marine women and men to assess whether the defining characteristics of a 300 Marine were consistent across sexes.

**TABLE 1:** US Marine Corps fitness test event requirements to earn maximum 100 points by sex and age group.

		Physical fitness test (PFT)				Combat fitness test (CFT)		
Sex	Age group	PU (n)	AC* (n)	PL (s)	3MR (s)	MTC (s)	AL (n)	MANUF (s)
Women	17–20	7	100	225	1,260	199	66	175
	21–25	11	105	225	1,260	193	74	165
	26–30	12	110	225	1,260	190	75	162
	31–35	11	105	225	1,260	192	72	169
	36–40	10	105	225	1,260	198	70	174
	41–45	8	100	225	1,290	205	62	178
	46–50	6	100	225	1,320	219	53	215
	51+	4	100	225	1,350	235	44	224
Men	17–20	20	105	225	1,080	160	106	127
	21–25	23	110	225	1,080	158	115	124
	26–30	23	115	225	1,080	159	116	125
	31–35	23	115	225	1,080	162	120	130
	36–40	21	110	225	1,080	165	110	136
	41–45	20	105	225	1,110	172	106	143
	46–50	19	100	225	1,140	181	100	160
	51+	18	100	225	1,170	185	95	172

*, discontinued event; 3 MR, three-mile run; AC, abdominal crunch; AL, ammunition lift; MANUF, maneuver-under-fire; MTC, movement-to-contact; PL, plank; PU, pull-up.

### Participants

United States Marine women and men were recruited as part of a body composition survey of the USMC (Potter et al., 2022c) from four locations: The Basic School (TBS) at Marine Corps Base Quantico, VA; active-duty Marines within the National Capital Region (NCR); Camp Lejeune, NC (CLNC); and Camp Pendleton, CA (CPEN). Out of the original sample of 2,175 volunteer Marines, we included those that: 1) achieved a passing score (150 or higher) on both the PFT and CFT; 2) self-reported data for all PFT and CFT events; 3) elected to complete the pull-up event (instead of the push-up option) and completed the three-mile run event; and 4) completed dual-energy x-ray absorptiometry (DXA) testing. The final sample consisted of 497 women (age, 29 ± 7 years; height 1.63 ± 0.07 m; body mass, 67.4 ± 8.4 kg) and 1,224 men (age, 30 ± 8 years; height 1.77 ± 0.07 m; body mass, 86.1 ± 11.1 kg). All research participants provided their written informed consent before study data collection. This study was approved by the US Army Medical Research and Development Command, Fort Detrick, MD, and the USMC institutional review boards (protocol M10873).

### Physical fitness test (PFT)

The USMC PFT consists of three events performed in any order during one ≤2 h session: pull-up/push-up; abdominal crunch/plank; and three-mile run (Keefer and Debeliso, 2020; Office of the Commandant of the Marine Corps, 2022). Marines can either perform the pull-up event for up to 100 pts or the push-up for a maximum of 70 pts. The abdominal crunch event was replaced by the timed plank after 31 December 2022. The plank is the only event with a single scoring table for Marine women and men of all age groups. The time required to score a perfect 100 on the plank was 260 s during this study but has since been reduced to 225 s (effective date 1 January 2022). The three-mile run is a timed event completed over a “out and back” or a wide loop three-mile (∼4.83 km) course without multiple sharp turns. Marines are required to wear the USMC approved green-on-green T-shirt, shorts, and running shoes for all PFT events.

### Combat fitness test (CFT)

**FIGURE 1:**
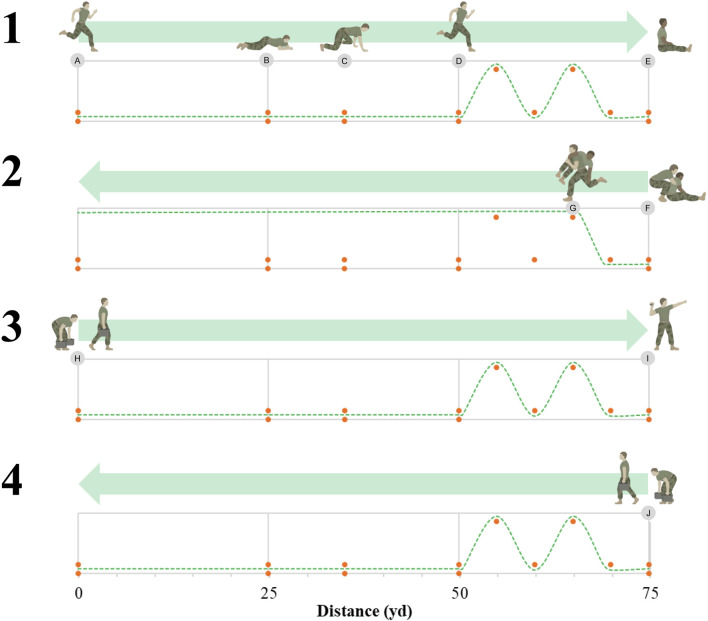
Layout of the maneuver-under-fire (MANUF) Combat Fitness Test (CFT) event. A, sprint 25 yd; B, high crawl 10 yd; C, modified high crawl 15 yd; D, zigzag sprint 25 yd; E, casualty; F, casualty drag 10 yd; G, fireman’s carry 65 yd; H, pickup ammo cans and carry 75 yd; I, grenade toss and 5 pushups; J, pickup ammo cans and carry 75 yd.

The USMC CFT also consists of three events performed in the following order during one ≤2 h session: movement-to-contact (MTC), ammunition lift (AL), and the maneuver-under-fire (MANUF) (Keefer and Debeliso, 2020; Office of the Commandant of the Marine Corps, 2022). The MTC is a timed 880 yd (∼804.7 m) run performed on either a track or measured surface with wide turnaround points and no sharp turns. The AL requires Marines to lift a 30 lb (∼13.6 kg) M2A1 5.56 mm ammunition can from shoulder height to overhead for as many repetitions as possible within a 2 min time limit. The MANUF is a timed 300 yd shuttle run that includes a variety of combat-related tasks including: sprint; high crawl; modified high crawl; diagonal run; casualty drag; fireman’s carry; ammunition can carry; and grenade toss (Figure 1). The casualty drag and fireman’s carry are completed with another Marine matched by height (±6 in, ∼15.2 cm) and weight (±10 lb, ∼4.5 kg). The authorized uniform for the CFT is the USMC Combat Utility Uniform and boots. Marines wear a green short-sleeve t-shirt for the AL event to ensure observation of elbow lock out for every repetition.

### Body composition

Standing height was measured using a standard stadiometer to the nearest 0.1 cm. Participants stood on a flat surface, with bare or stocking feet together, knees straight, with the head, shoulder blades, buttocks, and heels in contact with the stadiometer. Body mass was measured using a calibrated electronic scale to the nearest 0.1 kg. Body composition was assessed via dual-energy x-ray absorptiometry (DXA) (iDXA, GE Lunar, Madison, Wisconsin, United States). Participants laid supine within the outlined assessment area of the DXA table for a ∼10 min whole-body scan.

**FIGURE 2:**
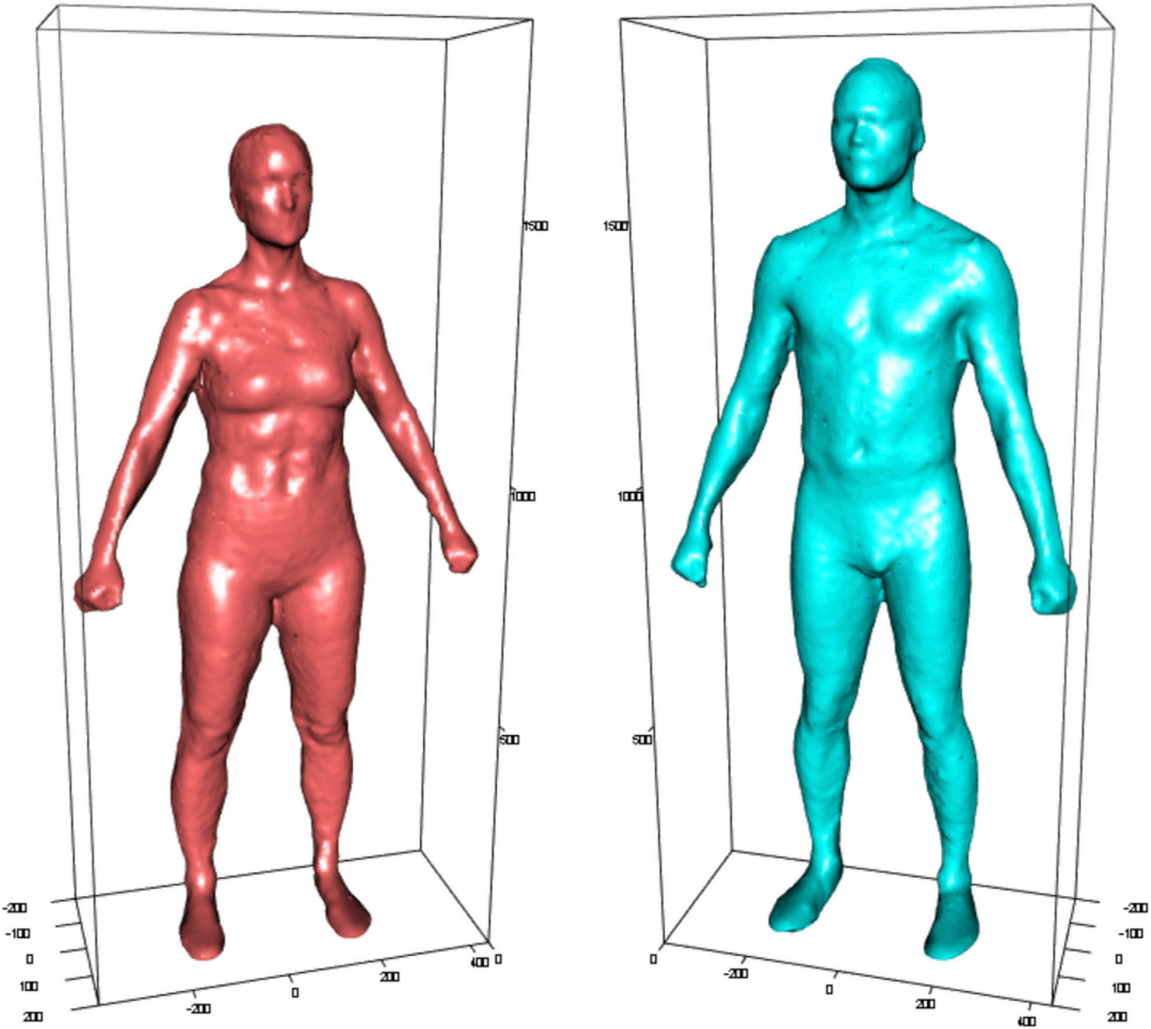
Three-dimensional body surface scans of the typical 300 US Marine woman and man (measurements in mm).

To provide a visual depiction of a typical 300 Marine woman and man, three-dimensional body surface scans were measured from a subset of the sample (*n* = 18; 10 women, 8 men) using a Size Stream SS20 Booth Scanner (SS20; Size Stream; Cary, NC, United States). Participants stood relaxed but still, with arms straight and abducted from the body on the SS20 platform throughout the entire 15 s scan. All participants wore only form-fitting compression shorts with female participants also wearing a sports bra. For each sex, the typical 300 Marine was defined as the participant with the lowest sum of squared standardized differences from the mean of the following measurements: height; body mass; bone mineral content (BMC) mass; fat mass; lean mass; body mass index (BMI); and percent body fat. Figure 2 displays three-dimensional scans of the typical 300 Marine woman and man.

### Statistical analyses

Data were analyzed using R (Version 4.3.3; R Foundation for Statistical Computing; Vienna, Austria) (R Core Team, 2014) and reported as mean ± standard deviation (SD). The α-level for statistical significance was set to 0.05. For most variables, we tested for significant differences between the 300 and Other Marines in women and men separately using independent t-tests. We calculated standardized group mean differences using Hedges’ g (mean difference divided by pooled standard deviation) (Brydges, 2019) and used thresholds for interpreting trivial (<0.2), small (0.20–0.49) medium (0.50–0.79), and large (≥0.80) effect sizes (Berjisian et al., 2022). Since all 300 Marines held the plank for the maximum time (260 s) and had the same 300 score on both their PFT and CFT scores, we instead evaluated between-group differences for these variables using one-sided t-tests. Specifically, we tested whether the Other Marines had plank times significantly less than 260 s and if their PFT and CFT scores were each significantly less than 300. We also evaluated whether standardized group mean differences were correlated between women and men across all measurements based on the Pearson correlation coefficient (r) with 95% confidence interval (95% CI). We used the following threshold ranges to interpret the correlation coefficient: negligible (0.00–0.10); weak (0.10–0.39); moderate (0.40–0.69); strong (0.70–0.89); and very strong (0.90–1.00) (Schober et al., 2018).

## Results

**FIGURE 3:**
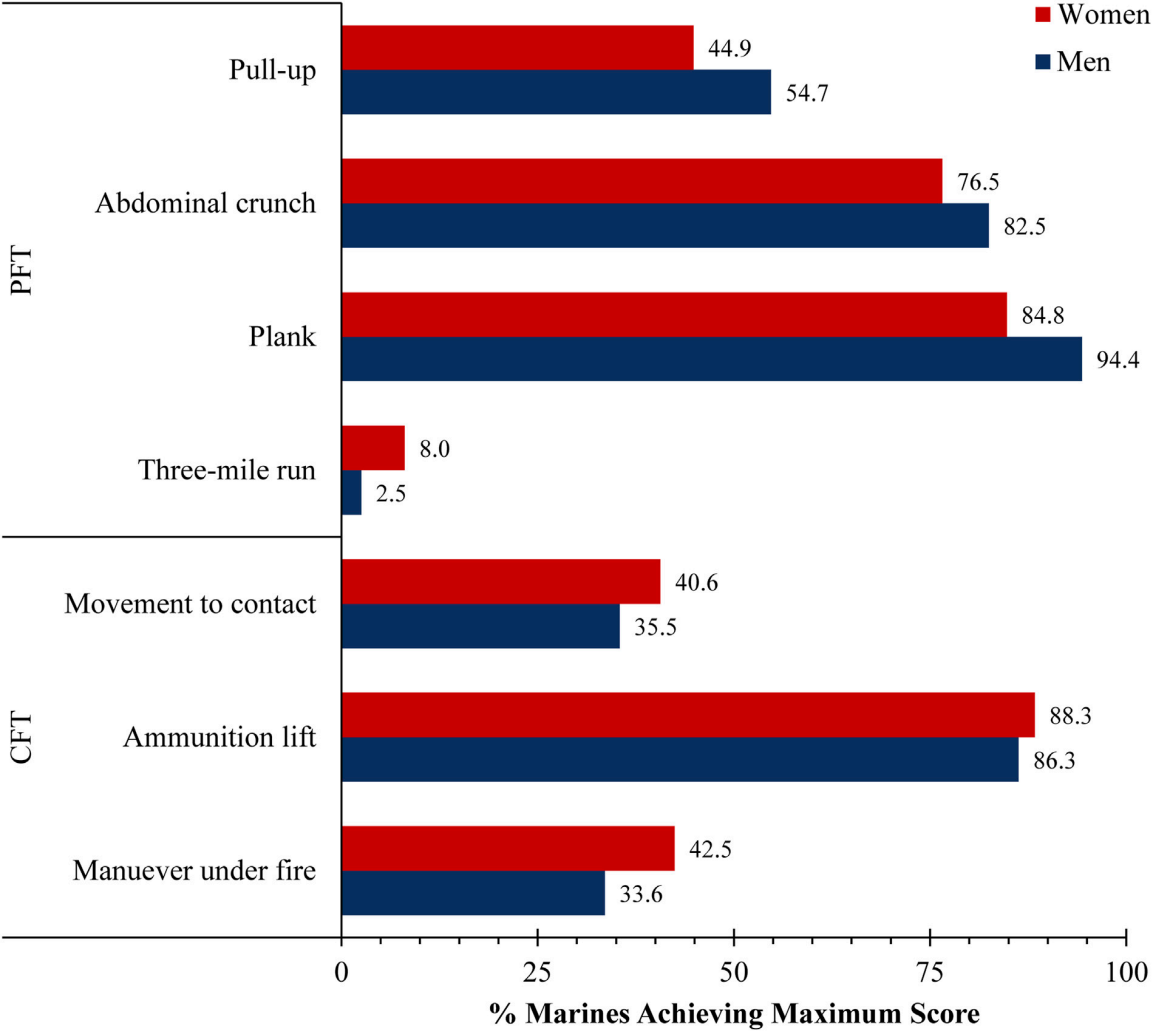
Percentage of Marines achieving maximum score on individual Physical Fitness Test (PFT) and Combat Fitness Test (CFT) events.

Only forty-three study participants (2.5% of the sample) earned 300 scores on both their PFT and CFT to qualify as a 300 Marine, including twenty-one women (4.2%) and twenty-two men (1.8%). Marines were far less likely to earn a maximum 100 score on the three-mile run than any other event (Figure 3). The 1,679 Other Marines earned significantly less than 300 points on the PFT (Women, 263 ± 24; Men, 264 ± 24) and CFT (Women, 283 ± 18; Men, 279 ± 21) (*p* < 0.001 for each). Age was not significantly different between the 300 and Other Marines in women (300, 29 ± 6 years; Other, 29 ± 7 years; Hedges’ g, 0.02, 95% CI [−0.41, 0.46]; *p* = 0.917) or men (300, 31 ± 10 years; Other, 30 ± 8 years; Hedges’ g, 0.14; 95% CI [−0.28, 0.56]; *p* = 0.517).

Compared to their peers, the 300 Marine women performed significantly more pull-ups (Hedges’ g, 1.12; 95% CI [0.67, 1.56]), and crunches (Hedges’ g, 0.84; 95% CI [0.35, 1.33]), with significantly lower times for the three-mile run (Hedges’ g, −2.30; 95% CI [−2.76, −1.84]), MANUF (Hedges’ g, −1.17; 95% CI [−1.62, −0.73]), and MTC (Hedges’ g, −1.33; 95% CI [−1.77, −0.88])) (*p* < 0.001 for each) (Table 2). Ammunition lift was not significantly different between the 300 and Other Marine women (Hedges’ g, 0.38; 95% CI [−0.05, 0.82]), *p* = 0.085). The 300 Marine men performed significantly more pull-ups (Hedges’ g, 0.62; 95% CI [0.20, 1.04]), *p* = 0.004) with significantly lower times for the three-mile run (Hedges’ g, −1.92; 95% CI [−2.35, −1.49]), MANUF (Hedges’ g, −0.85; 95% CI [−1.27, −0.43]), and MTC (Hedges’ g, −1.07; 95% CI [−1.50, −0.65]), (*p* < 0.001 for each). There were no significant differences in abdominal crunch (Hedges’ g, 0.18; 95% CI [−0.27, 0.64]; *p* = 0.434) or AL (Hedges’ g, 0.24; 95% CI [−0.18, 0.67]; *p* = 0.256). Plank times were significantly less than the 260 s maximum time for both the Other Marine women and men (*p* < 0.001 for each).

**TABLE 2:** Comparison of Physical Fitness Test (PFT) and Combat Fitness Test (CFT) performance between the 300 and Other Marines (Mean ± SD).

		Group	Women	Men
PFT	Pull-up (n)	300	13 ± 4*	23 ± 2*
		Other	9 ± 3	20 ± 5
	Abdominal crunch (n)	300	115 ± 15*	114 ± 9
		Other	106 ± 10	113 ± 9
	Plank (s)	300	260 ± 0*	260 ± 0*
		Other	243 ± 33	252 ± 20
	Three-mile run (s)	300	1,179 ± 108*	1,080 ± 91*
		Other	1,487 ± 135	1,337 ± 134
CFT	Movement-to-contact (s)	300	172 ± 16*	149 ± 16*
		Other	203 ± 24	171 ± 20
	Ammunition lift (n)	300	83 ± 19	119 ± 12
		Other	78 ± 13	117 ± 9
	Maneuver-under-fire (s)	300	149 ± 14*	123 ± 17*
		Other	176 ± 23	139 ± 19

*, significantly different between the 300 and Other Marines (*p* < 0.05).

The 300 Marine women had significantly higher lean mass (Hedges’ g, 0.46; 95% CI [0.02, 0.89]; *p* = 0.041) with significantly lower fat mass (Hedges’ g, −1.11; 95% CI [−1.55, −0.67]), BMI (Hedges’ g, −0.74; 95% CI [−1.18, −0.30]), and percent body fat (Hedges’ g, −1.34; 95% CI [−1.79, −0.90]) (*p* < 0.001 for each) (Table 3). There were no significant differences in height (Hedges’ g, 0.37; 95% CI [−0.07, 0.80]; *p* = 0.102), body mass (Hedges’ g, −0.38; 95% CI [−0.82, 0.05]; *p* = 0.085), and BMC mass (Hedges’ g, 0.15; 95% CI [−0.29, 0.59]; *p* = 0.502). The 300 Marine men had significantly lower body mass (Hedges’ g, −0.48; 95% CI [−0.91, −0.06]; *p* = 0.025) as well as fat mass (Hedges’ g, −0.94; 95% CI [−1.36, −0.51]), BMI (Hedges’ g, −0.80; 95% CI [−1.23, −0.38]), and percent body fat (Hedges’ g, −0.99; 95% CI [−1.41, −0.57]) (*p* < 0.001 for each). There were no significant differences in height (Hedges’ g, 0.35; 95% CI [−0.07, 0.78]; *p* = 0.101), BMC mass (Hedges’ g, −0.02; 95% CI [−0.44, 0.40]; *p* = 0.915), and lean mass (Hedges’ g, 0.11; 95% CI [−0.31, 0.53]; *p* = 0.618).

**TABLE 3:** Body composition of the 300 Marines measured by dual-energy x-ray absorptiometry (DXA) (Mean ± SD).

	Group		Women	Men
Height (cm)	300		166 ± 6	179 ± 5
	Other		163 ± 7	177 ± 7
Body mass (kg)	300		64.3 ± 5.9	80.8 ± 7.1*
	Other		67.5 ± 8.5	86.2 ± 11.1
BMC mass (kg)	300		2.6 ± 0.3	3.3 ± 0.4
	Other		2.6 ± 0.3	3.3 ± 0.4
Fat mass (kg)	300		13.9 ± 4.1*	13.0 ± 4.3*
	Other		19.7 ± 5.3	19.2 ± 6.7
Lean mass (kg)	300		47.7 ± 3.8*	64.6 ± 5.9
	Other		45.2 ± 5.6	63.7 ± 7.9
Body mass index (kg/m^2^)	300		23.4 ± 1.8*	25.2 ± 1.8*
	Other		25.4 ± 2.6	27.6 ± 3.0
Percent body fat (%)	300		21.5 ± 4.8*	15.9 ± 4.8*
	Other		29.0 ± 5.6	21.9 ± 6.1

*, significantly different between the 300 and Other Marines (*p* < 0.05); BMC, bone mineral content.

**FIGURE 4:**
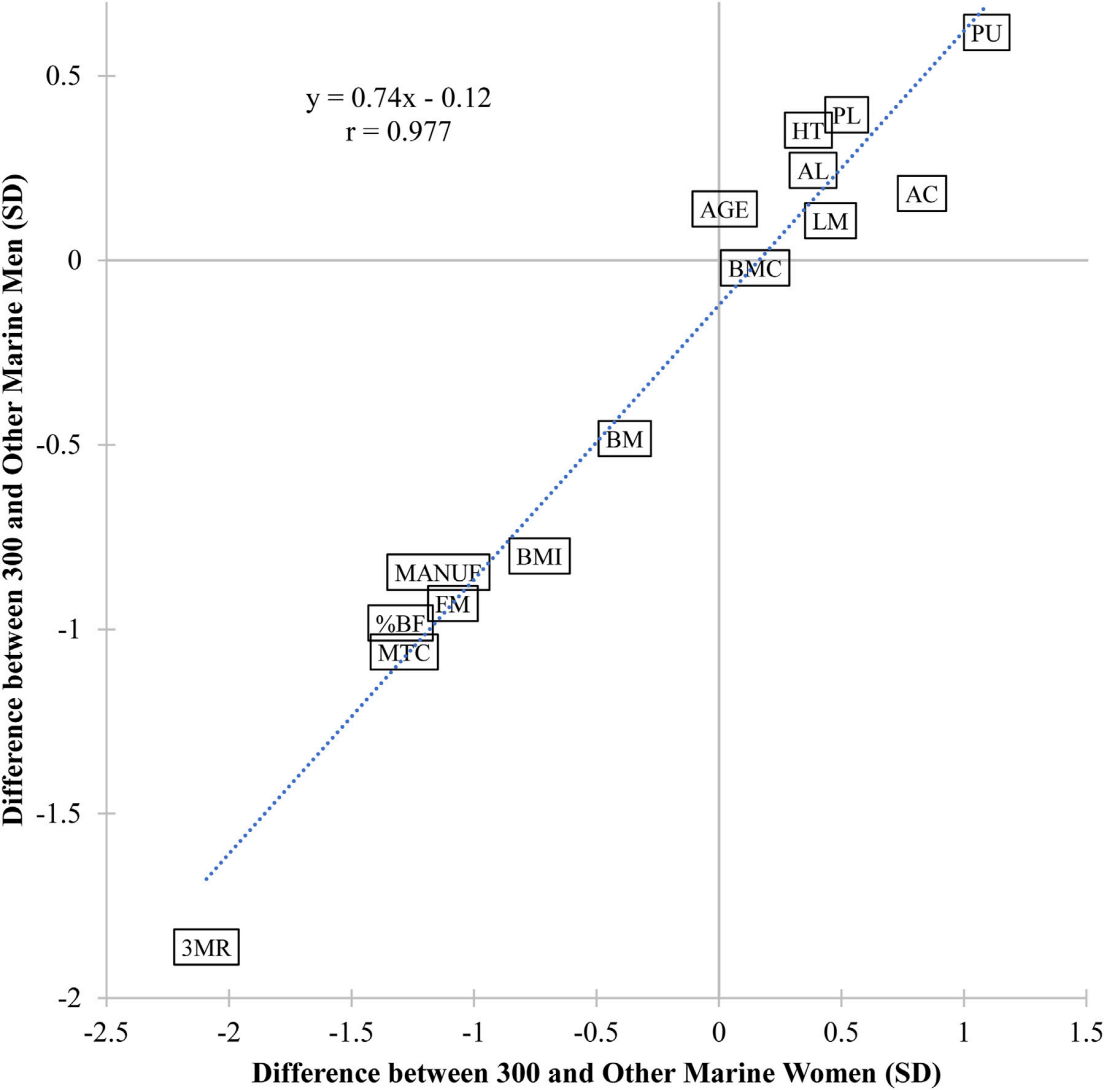
Association of standardized group mean differences between US Marine women and men. %BF, percent body fat; 3 MR, three-mile run; AGE, age; AC, abdominal crunch; AL, ammunition lift; BM, body mass; BMC, bone mineral content mass; BMI, body mass index; FM, fat mass; HT, height; LM, lean mass; MANUF, maneuver-under-fire; MTC, movement-to-contact; PL, plank; PU, pull-up; r, Pearson’s correlation coefficient; SD, standard deviation.

Overall, standardized mean group differences had a very strong correlation between women and men (r = 0.977; 95% CI [0.931, 0.993]) (Figure 4). The largest differences were observed in the three-mile run, which was 2.09 and 1.86 SD lower in 300 Marine women and men than their peers respectively, further evidence for the association of defining characteristics of a 300 Marine between sexes.

## Discussion

This is the first study focused on the elite population of “300 Marines” that achieve perfect scores on their annual fitness tests. In terms of physical and combat fitness, our analyses demonstrate that performance on maneuverability events (three-mile run, MTC, MANUF) is what separates 300 Marines from their peers on these tests. The PFT and CFT carry high importance in the Marine Corps, counting heavily towards career progression and used to evaluate deployment readiness. These tests have been refined over many years since the 1950s and are viewed as reflecting a level of physical capability for operational performance (Keefer and Debeliso, 2020).

The body composition associated with top performance scores is informative about the nature of the tests. Compared to all other Marines, men and women scoring 300 had lower fat mass, BMI, and percent body fat than their peers. These are expected attributes that would be associated with better running and maneuver whereas greater emphasis on strength performance would be associated with greater mass, including less penalty for fat mass. Altogether, relative force production would appear to be an important trait for optimal Marine fitness, given its advantages for both strength and maneuverability capabilities. However, this needs to be verified by a dedicated research investigation on this population. The lower BMI of the 300 Marine women was explained by greater height and lower fat mass compared to their peers while the 300 Marine men had similar lean mass but lower body mass (i.e., lighter) due to lower fat mass, suggesting that maintenance of low body fat percentage is important for USMC fitness test performance, or that regular physical training and energy expenditure that results in high test scores also reduces stored fat. As the standardized group mean differences in fitness and anthropometric assessments were very strongly correlated between 300 Marine women and men (r = 0.977), we determined that the defining characteristics of a 300 Marine converge and are consistent across sexes (Figure 4). It is also noteworthy that Marine women can perform pull-ups, which distinguishes them in general from most other military men and women (Potter et al., 2023). These data provide an important baseline for evaluation of future changes in physical readiness testing and assessment of future populations as well as highlight the importance of strength in tactical populations.

The balance between aerobic and resistance exercise has a significant influence on training adaptations, with a high volume of aerobic exercise attenuating the muscle volume and strength gains from resistance exercise (Santtila et al., 2009). Low percent body fat can occur through modest increases in lean mass as well as from high volume aerobic training. Although percent body fat is a poor predictor of performance because of the huge variation in body composition relative to physical performance, it is consistently lower in high fitness populations, reflecting the effects of regular physical training on relatively greater muscle mass and the high daily energy expenditure reducing fat.

It should also be noted that the average percent body fat of the “300” women were lower than the normative data for fit healthy women developed from Marine Lieutenants in the Basic School, while the average percent body fat of the “300” men was similar to fit healthy men in the same study (Potter et al., 2024). The low percent body fat in “300” women is similar to previously observed low percent body fat in women engaged in high-intensity interval training and reflects an increased lean mass not typically seen in other women or with other types of training. The USMC has a broad emphasis on High Intensity Tactical Training (HIIT) programs in their fitness facilities (Haddock et al., 2016), making this training available to all Marines. It may be noteworthy that the characteristics of this group of women are comparable to those reported for another group of elite women in a much smaller sample (McClung et al., 2022). That sample included one Marine graduate of the Infantry Officers Course along with a dozen soldier graduates of the Army Ranger course. These women also reported regular HITT-type training.

The importance of maneuverability to the 300 Marine is best exemplified by the paucity of individuals that achieve maximum scores on the three-mile run (women, 8.0%; men, 2.5%) (Figure 3). The maneuver-under-fire and movement-to-contact tests have high face validity for high priority basic rifleman skills, and it is noteworthy that the high performing “300” also stood out from other Marines by their ability on these tests (Figure 4). This might suggest that there is some correlation between strength and aerobic performance and performance on these two practical combat fitness tests. Abdominal crunch, timed plank, and ammunition lift are more generally tests of muscular endurance and were not distinguishing characteristics of the “300”, perhaps due to being less physically demanding than the other USMC fitness tests.

We did not have information on daily training of the individuals but hypothesize that the “300” train more intensively and regularly than their lower scoring peers. The training habits as well as other health and fitness behaviors of this exceptional group should be the focus of a follow-on study that includes training logs, wearable physiological monitoring of sleep and activity patterns, and nutritional and other health habits surveys. Identifying whether 300 Marines possess superior lower body force production capabilities, using an isometric mid-thigh pull test or similarly objective and reliable assessment (Comfort et al., 2019), is another important topic for future research. We have established characteristics of high scoring Marines but there is an underlying premise that the PFT and CFT are perfected test batteries that predict operational performance capabilities of Marines. This association with battlefield performance has not been objectively demonstrated but rather based on subject matter expertise. A future study of battle-hardened veteran Marines might better address this. Alternatively, characterization of Marines training with allies in annual deployment exercises might also serve as a test of the incumbent characteristics.

## Conclusion

The “300 Marines” that achieve the highest possible scores on their annual PFT and CFT are most distinguished in fitness from their peers by their maneuverability. These Marines have considerably less percent body fat than their sex-matched counterparts, with 300 Marine women possessing greater lean mass than their peers whereas 300 Marine men are relatively lighter in weight. Overall, the defining characteristics of a 300 Marine are consistent across sexes, suggesting a similar set of desirable traits for elite performing women and men.

## Data Availability

The raw data supporting the conclusion of this article will be made available by the authors, without undue reservation.
